# Talkitt: toward a new instrument based on artificial intelligence for augmentative and alternative communication in children with down syndrome

**DOI:** 10.3389/fpsyg.2023.1176683

**Published:** 2023-06-06

**Authors:** Floriana Costanzo, Elisa Fucà, Cristina Caciolo, Deborah Ruà, Sara Smolley, Danny Weissberg, Stefano Vicari

**Affiliations:** ^1^Child and Adolescent Neuropsychiatry Unit, Bambino Gesù Children’s Hospital, IRCCS, Rome, Italy; ^2^Voiceitt Ltd., Buffalo, NY, United States; ^3^Department of Life Science and Public Health, Catholic University of the Sacred Heart, Rome, Italy

**Keywords:** language, machine learning, trisomy 21, digital application, speech

## Abstract

**Introduction:**

Individuals with Down syndrome (DS) often exhibit a severe speech impairment, with important consequences on language intelligibility. For these cases, the use of Augmentative Alternative Communication instruments, that increase an individual’s communication abilities, becomes crucial. Talkitt is a mobile application created by Voiceitt Company, exploiting speech recognition technology and artificial intelligence models to translate in real-time unintelligible sounds into clear words, allowing individuals with language production impairment to verbally communicate in real-time.

**Methods:**

The study evaluated the usability and satisfaction related to the Talkitt application use, as well as effects on adapted behavior and communication, of participants with DS. A final number of 23 individuals with DS, aged 5.54 to 28.9 years, participated in this study and completed 6 months of training. The application was trained to consistently recognize at least 20 different unintelligible words (e.g., nouns and/or short phrases)/person.

**Results:**

Results revealed good usability and high levels of satisfaction related to the application use. Moreover, we registered improvement in linguistic abilities, particularly naming.

**Discussion:**

These results paves the road for a potential role of Talkitt application as a supportive and rehabilitative tool for DS.

## 1. Introduction

Down syndrome (DS) is the most common genetic cause of intellectual impairment, estimated to occur once in approximately 1,000 births (Grimm et al., 2021). A high variability in the degree of cognitive impairment, ranging from profound to borderline intellectual functioning, is observed (Roizen, 2002; Vicari et al., 2013). Individuals with DS exhibit a neuropsychological profile characterized by weaknesses in the processing of verbal information associated with relatively spared visual information; moreover, they frequently exhibit delays in language development with receptive abilities usually more preserved than expressive abilities (Grieco et al., 2015). In particular, comprehension is usually related to the developmental stage, whereas expressive language quality is impaired in both vocabulary and syntax; for instance, it has been shown that syntax delays in individuals with DS are beyond expectations for cognitive level (Chapman and Hesketh, 2001; Eadie et al., 2002; Price et al., 2008). Speech production in DS is highly impaired: frequent sound errors with both protracted use of developmental phonological processes, such as final consonant deletion, and atypical phonological processes, presenting in association with inconsistent whole-word productions are frequently observed (Dodd and Thompson, 2001; Roberts et al., 2005; Cleland et al., 2010). Moreover, individuals with DS frequently omit words belonging to some grammatical categories such as prepositions or articles (Grieco et al., 2015). Altogether, these disorders compromise speech intelligibility. Over the years, a great number of risk factors have been identified for reduced speech intelligibility in DS, such as neuropsychological factors (e.g., short-term memory deficits), peculiar craniofacial features causing variations in laryngeal and resonator properties of speech, and hearing difficulties (Jarrold and Baddeley, 1997; Kent and Vorperian, 2013). However, there is no widely-accepted explanatory construct to sustain interventions for reduced intelligibility in DS (Faught and Conners, 2019; Wilson et al., 2019).

Overall, limited speech intelligibility represents a major issue in DS. Indeed, 95% of parents declared to be concerned about their child’s ability to be understood (Kumin, 1994), and it has been documented that unintelligible speech in DS is a severer problem also in comparison with other intellectual disabilities (Rosin et al., 1988; Abbeduto and Murphy, 2004). Indeed, more than half of adolescents with DS have a hard time making themselves understood by anyone other than caregivers (Van Gameren-Oosterom et al., 2013). Moreover, important difficulties with morphosyntax and speech intelligibility can continue into adult age, hampering full participation in community life and independent living (Chapman and Hesketh, 2000). Therefore, supporting communication in DS represents a crucial aspect to promote socialization in such a population (Rodenbusch et al., 2013; Wilkinson and Finestack, 2020), supporting an amelioration in adaptive abilities and overall quality of life. Indeed, adaptive behavior includes conceptual, social, and practical skills required to function in everyday lives (Rapley, 2004). Communication skills, both comprehension and language production, are key component of social adaptive behaviors, allowing the individual to actively participate to the social environment he/she is included in.

Thus, since communication impairment plays a critical role in the development and social engagement of children with DS, it is fundamental to provide them with support aiding interaction processes. In particular, Augmentative and Alternative Communication (ACC) provides support to individuals with intellectual disabilities and complex communication needs (Beukelman and Light, 2020).

AAC is an assistive technology dedicated to patients with communication difficulties, which comprises several kinds of communication (other than verbal) and promotes all kinds of augmentative aids. According to the American Speech–Language–Hearing Association (American Speech-Language-Hearing Association, 2019), it is possible to classify AAC systems by distinguishing between aided and unaided systems. Unaided AAC systems involve the use of some parts of the body with communicative purposes, such as pointing, gestures, and facial expressions. Aided AAC systems involve the use of low-technology, such as symbol-based communication boards or books, or mid- and high-technology aids, such as speech-generating devices or electronic equipment (e.g., speech-generating devices, tablets with AAC applications; American Speech-Language-Hearing Association, 2019). AAC technology is evolving very rapidly. In the last years, AAC is taking advantage of a wide range of systems exploiting machine learning (ML) models to process and generate outputs by optimizing word prediction models and speech recognition algorithms; through ML, AAC systems can produce outputs in electronic digitized or synthesized speech (Elsahar et al., 2019). The relative affordability of mobile devices such as smartphones and tablets, associated with their portability and social acceptability (Still et al., 2014) makes high-technology AAC systems particularly suitable for individuals with developmental disabilities. Accordingly, some evidence on the preference of individuals with neurodevelopmental disorders for high-technology AAC has been reported (Ganz et al., 2013; Lorah et al., 2013; Couper et al., 2014). In their meta-analysis, Ganz et al. (2017) found low to moderate positive effects on social-communication outcomes for high-tech AAC use by individuals with intellectual and developmental disabilities throughout all school years. In particular, the meta-analysis reported that, despite some research demonstrating weak effects of AAC applications for this population, individual studies were significantly effective. The authors also considered a number of possible moderators to identify for whom and under what circumstances high-tech AAC implementation might be more or less effective. Results suggested AAC efficacy being independent from the implementer (i.e., researcher vs. natural communication partner), intervention context (i.e., natural vs. didactic contexts), behavioral strategies, age of participants, and communicative functions.

A recent systematic revision of the literature identified 12 AAC instruments for which some evidence of efficacy for people with ASD has been reported (Barbosa et al., 2018). In particular, systems such as Picture Exchange Communication System and Picture communication symbols seem to increase the interaction between individuals with DS and their peers, contributing to improving their quality of life and self-esteem (Barbosa et al., 2018). However, the authors highlighted the need for further well-designed studies investigating the effectiveness of various AAC devices to promote communication, socialization, and language abilities in DS. In particular, research investigating the effectiveness of high-technology AAC for DS is highly needed.

The present study was part of a broader project entitled “Speech recognition technology to enable people with Speech disabilities to communicate freely,” funded by the Horizon 2020 program and coordinated by Voiceitt, a speech-recognition technology company. The Voiceitt team developed a customizable speech recognition system, the Talkitt application, a software application that translates unintelligible sounds into clear speech in real-time using a speech recognition algorithm. The purpose of the broader project was to optimize the Talkitt Basic application and validate it in multi-country trials on different populations. The optimization concerned the algorithm increased accuracy and recognition rates from 75% to 90%, overcoming error-free calibration and increasing discrimination, estimation of noise conditions and system stability. The validation in multi-country trials was aimed to demonstrate the applicability of the application (usability and satisfaction) in different environments and countries (Israel, United Kingdom, Spain, and Italy) with different languages, translating the user interface to ensure language independent use. The validation in different populations was aimed to demonstrate the applicability of the app (usability and satisfaction) and the impact on adapted behavior in people with different ages, diagnoses, and severities of speech disability (Acquired and developmental diseases, Traumatic brain injury, Stroke, Autism Spectrum Disorder, DS). Within the broader project, the present study aimed at training and demonstrating the applicability of the Talkitt Basic application in individuals with DS of Italian language. In particular, the present study aimed at training the artificial intelligence system dedicated to the recognition of vocal tracks spoken by individuals with SD with poorly intelligible language, and to the return of the correct interpretation of the audio track (audio reproduction of the word in real-time). The training of the system was necessary to build up a predictive mathematical model, based on ML, optimizing the speech recognition of 20 different unintelligible words/persons in our sample of children and adolescents with DS. To demonstrate the applicability, we evaluated the caregivers’ satisfaction in using the Talkitt application and explored the impact of the use of the device on adaptive behavior. Communication abilities were also evaluated. The present study describes the results of the applicability of the application in our sample of individuals with DS, while the results on the algorithm improvement will be analyzed as part of the broader project results and will be described elsewhere.

## 2. Materials and methods

### 2.1. Participants

Participants with DS were recruited at the Child and Adolescent Neuropsychiatry Unit of a Children’s Hospital in Rome. Italian was the primary language spoken at home for all participants. Inclusion criteria were as follows: diagnosis of DS confirmed by genetic testing; chronological age > 5 years; mental age ≥ 3.6 years; scores < 2 SD at the articulation subtest of Battery for Language Assessment in children aged 4 to 12 (Batteria di Valutazione Linguistica—BVL: 4–12; Marini et al., 2015). All participants were required to communicate verbally using a consistent language, but exhibiting moderate to severe phonological alterations; be comprehensible to closest relatives, at least in part, exhibiting consistent speech sounds; and having performed an otolaryngological examination that ruled out sensorineural hearing impairment and/or prescription hearing aids. Non-speaking individuals and youths with mild phonological alterations in expressive language (i.e., scores above −2 SD at the articulation subtest of Battery for Language Assessment) were excluded from the study. Participants’ caregivers were also required to have an e-mail address to register with the Talkitt application and receive electronic communications while using it. Non-speaking individuals and youths with mild phonological alterations in expressive language were excluded from the study. We considered a language “consistent” when the same sequence of phonemes is produced each time it is uttered in the same context (e.g., picture naming). Phonetic variation in the production of a phoneme, captured by phonetic transcription, was not considered inconsistent. For example, [/a’mɛlla/] and [/a:a’mɛlla/], instead of [/kara’mɛlla/], *caramella* (candy), are two uncorrected forms but are not phonologically inconsistent, as the word is produced without a phonemic contrast each time (Bürki, 2018). Conversely, we considered a language as “inconsistent” when there are idiosyncratic words, words containing more than three phonological variations, the use of contrasting phonology, and the preference for one sound (e.g., theism). The assessment was made by an experienced clinical speech pathologist.

Thirty-four individuals with DS, aged 5.54 to 28.9 years (M = 11.25; SD = 5.19), participated in this study. Twenty-six participants were male and 8 were female. Intelligent Quotient (IQ) ranged between 43 and 69 (M = 56.37, SD = 8.13). Their diagnosis was molecularly confirmed by genetic test and all showed 21 free trisomy. All participants presented consistent language with different phonological and morphosyntactic difficulties and unintelligible speech. Out of the 34 participants, 23 (18 M, 5F) completed all the testing and the follow-ups, their average age was 9.44 years (ranging between 5.54 and 28.9 years; SD = 5.15) and their average IQ was 59.78 (ranging between 43 and 69; SD = 5.15).

### 2.2. Method

#### 2.2.1. Talkitt application

Talkitt application is based on Voiceitt software that translates unintelligible sounds into clear speech in real-time using its proprietary speech recognition algorithm that recognizes unintelligible speech, and a large voice database of recordings from people with non-standard speech due to a variety of underlying conditions and disabilities.

The conceptual basis for the Voiceitt algorithms comes from the experiences of people with speech disabilities to date. It was observed that while people with these impairments struggle to be understood by outsiders, they are often understood with ease by family, friends, or caregivers who have learned how to adapt to their unique pattern of speech or prosody. From these observations, Voiceitt has been able to construct the Voiceitt algorithm to replicate this motion and recognize unintelligible speech. The innovation is in the recognition of unintelligible speech that requires powerful algorithms able to differentiate between indiscriminate sounds that are otherwise unintelligible to the human ear and standard speech systems.

Recognizing the complexity and the unique needs and characteristics of the users it serves, the core technology that forms Voiceitt’s multi-layered solution is built upon main automatic speech recognition (ASR) architectures adapting state-of-the-art ASR techniques to recognize unintelligible speech:

Voiceitt Discrete ASR (DASR)—Voiceitt first-generation technology, called “discrete” speech recognition, is a customizable, language-independent, on-device ASR engine designed to suit highly unintelligible speech—i.e., what has been pre-calibrated by each user.

**Figure 1:**
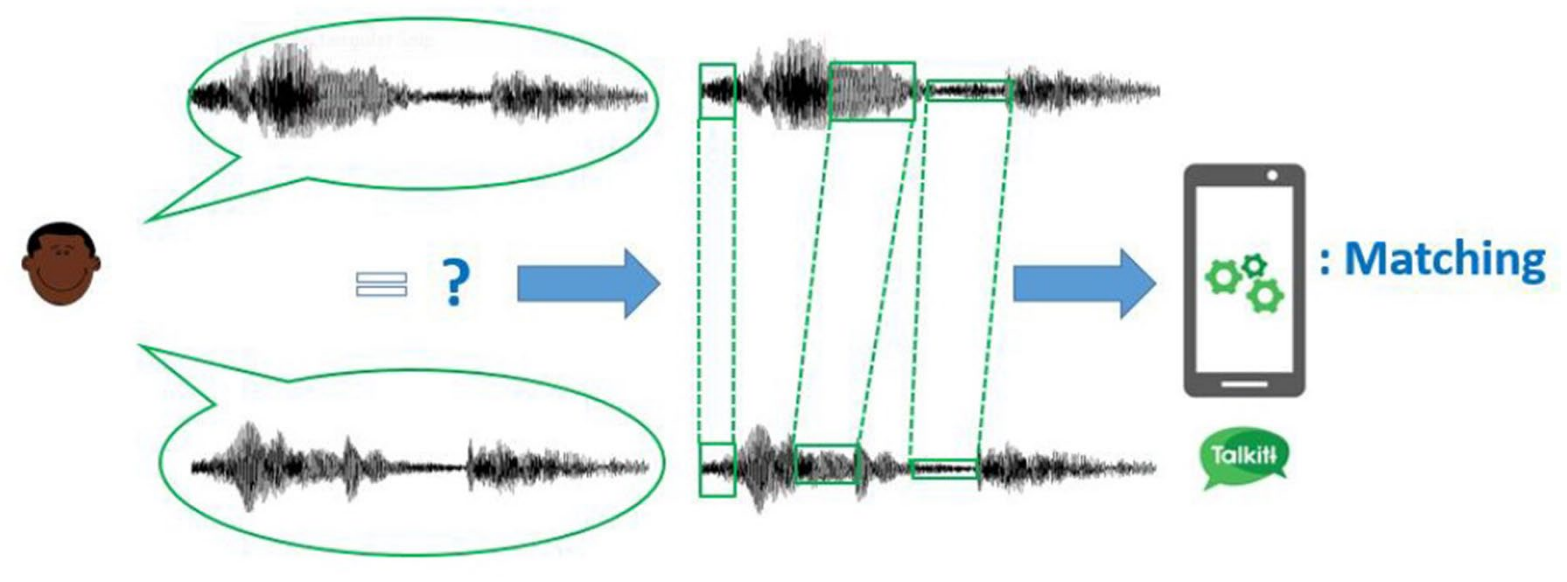
Illustration of how the Talkitt algorithm identifies the patterns of sound unique to the unintelligible speech user and use frame matching to map those consistencies to standard speech and the recorded sounds, thus successfully translating unintelligible speech. This figure is reproduced with permission from the Voicett company.

The Voiceitt iOS “discrete” application offers a personalized speech bank, or “dictionary.” It requires the user to create and maintain a unique collection of their own specific words, phrases, and utterances. It is limited to the number of words or short phrases saved in the user’s personalized dictionary, which they have chosen. Using the Voiceitt iOS application, the user will record words or phrases during the calibration phase to be stored in their personal speech bank and enable pattern matching based on prosody features. The more words added by a user, the greater level of recognition accuracy will be achieved. The application is limited to the communication content calibrated by the user, although she may pre-program an unlimited number of phrases into the dictionary. The user can also select the voice output, with the choice between adult-child and male–female voice outputs. Of note, the application does detect the differences in changes in the pronunciation of words or phrases. It tries to generalize the variations and map directly from a sound to a phrase/word and captures the speech variability intrinsically in the model. This requires the training set to be diverse so that it captures the typical variability (changes to pronunciations). The application also learns from the usage of the application. As a result, the examples that are used for training have increased variability over time. Therefore, during the on-boarding and training phase, participants actively “trained” the Artificial Intelligent system in recognizing selected words. Figure 1 shows how the algorithm identifies the patterns of sound unique to the unintelligible speech user.

Each individual user has a distinct phonetic inventory and adapting to their speech is similar to adapting the system for a new language. For each user, the larger collection of speech samples builds a better speech recognition model. The Talkitt algorithm is based on the estimation of speaker-dependent phonetic inventory by clustering similar sub-word linguistic units: upon launch of the application, an initialization requires the user to provide very small sample of recordings (five words, each repeated two times) which form clusters. The algorithm learns how the user says these specific words and recognizes them when spoken. In particular, the algorithm computes short-term signal energy of input each 20 ms over a window of 250 ms and, based on some pre-tuned threshold, it detects speech. If the computed energy is larger than a predefined threshold, start of a speech is declared; if it is the lower, end of speech is declared. As a unique user continues to use the application in this form, the algorithm steadily learns from the new recordings of the user that it receives.

**Figure 2:**
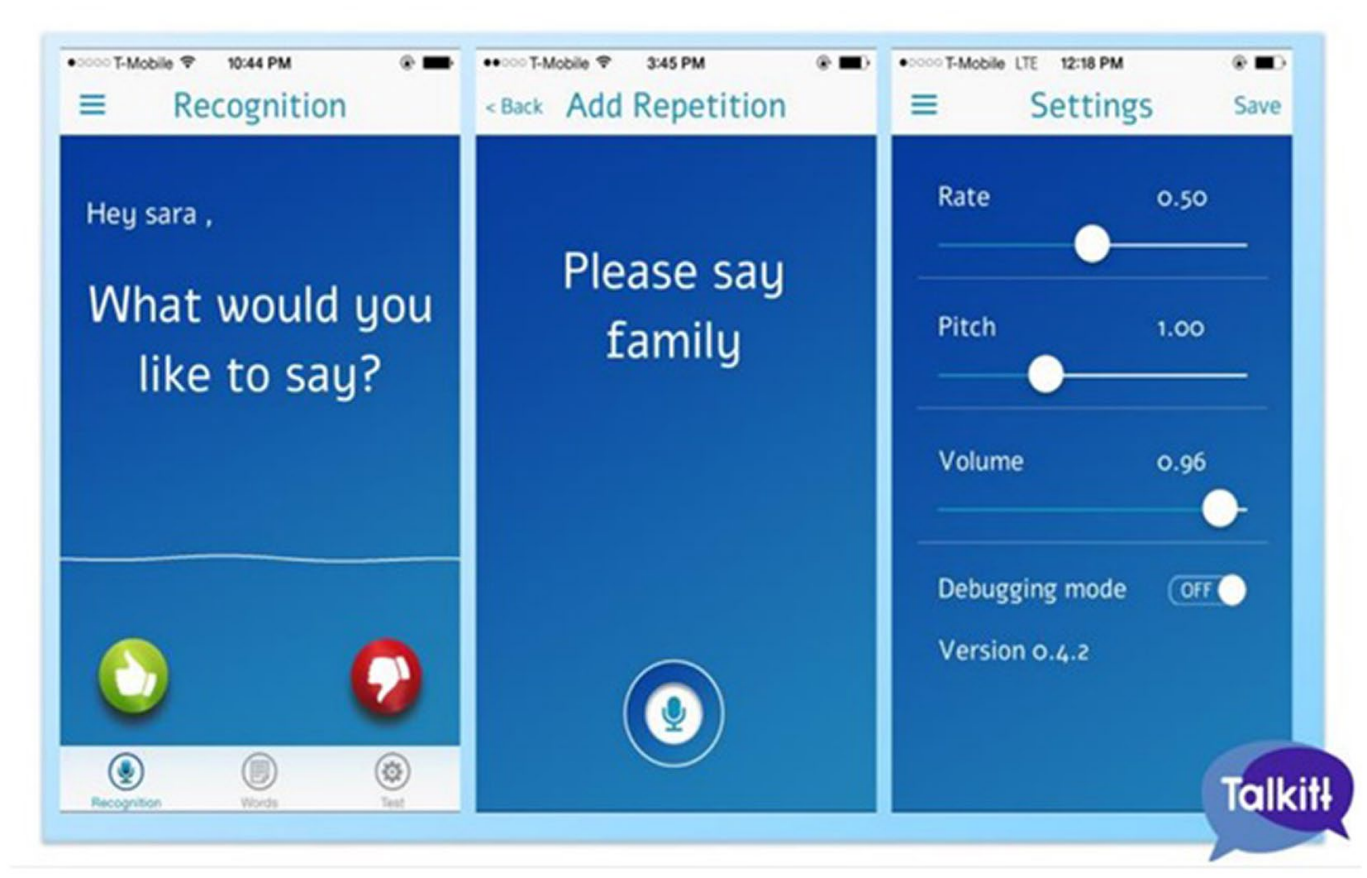
User interface design. The application is intuitive, requiring minimum or null support for usage. This figure is reproduced with permission from the Voicett company.

By collecting a larger number of recordings from any one user, the algorithm can run clustering methods on the phonetic characteristics and identify units of sound in the user’s unique speech style. This allows the mapping of these units in standard speech recognition and the application to a more extensive, unlimited vocabulary. Moreover, the user interface is intuitive, requiring minimum or null support for using it. See Figure 2 for user interface design.

#### 2.2.2. Procedure

##### 2.2.2.1. Enrollment, onboarding, and training

Enrollment. Participants were enrolled by requesting voluntary participation from families and children at the Child and Adolescent Neuropsychiatry Unit of a Children’s Hospital and by involving DS associations operating at the national level. Informed consent was obtained from parents/caregivers of children and adolescents along with informed consent to the direct voluntary participant of the study.

Onboarding and training. For each participant, in agreement with caregivers, we selected 3–5 poorly intelligible words (nouns and/or short phrases) frequently pronounced by the child within specific daily life contexts (named scenarios). Families were invited to register about 20 repetitions/word for the entire duration of the project (6 months). Participants were instructed to pronounce the selected word naturally, without emphasizing specific parts of the word, thus avoiding, for instance, vocal emphasis, lengthening of syllables. Vocal data resulting from the registration were uploaded to the Voiceitt database to develop and improve the mathematical model of vocal recognition for each participant. Subsequently, parents could independently carry out onboarding for new additional words.

Through of the Voiceitt “Ambassador” software (“dashboard”), we carried out daily online remote monitoring to verify the use of the Talkitt application by the participants and, if necessary, to provide suggestions for the improvement of the voice model of each user. In particular, we checked the number of daily recordings, and the quality of the audio tracks and performed cutting of the essential track to the Artificial Intelligent System, when required.

##### 2.2.2.2. Beta testing

Talkitt beta testing lasted for 6 months. It consisted of the daily use of the Talkitt device during the conversation. Participants were instructed to use the application daily (including the weekends) for 6 months, as much as possible within the scenarios chosen in the on-boarding and training phase. The mathematical model updated every time the patient pronounced the three words entered, recording the spoken vocal form (recording occurred only if the vocal form was recognized by the system). This training in the mathematical model made possible the continuous updating of the system of all the small modifications of the emitted vocal form. Researchers performed remote monitoring of the appropriateness of the vocal forms recorded by the system as corresponding to the words entered; they were able to accept or reject the inclusion of new recordings in the mathematical model of speech recognition of the initial three words. After obtaining a sufficient number of repetitions for each word (about 20), vocal forms of other words were recorded, up to about 20 words. The procedures for inserting the vocal forms into the mathematical model follow the abovementioned procedure. To have a variety of vocal samples and diversified confusing background noises, participants and caregivers were instructed to use the Talkitt device at various times of the day. During the 6 months, researchers were constantly in touch with engineers of the Voiceitt team to face possible issues. In the automatic phase, i.e., when the Artificial Intelligent System has now learned the unintelligible words of the child or teenager, the Talkitt application has reproduced the correct spoken vocal form. At the same time, to refine the training of the system, through the same Talkitt application, the child, the parent, or the child’s therapist could confirm whether the interpretation of the system was correct or not.

##### 2.2.2.3. Re-test

The intermediate re-test consisted of an application setting check to verify the technical functionality. The final re-test consisted of the administration of standardized tests administered during the enrollment phase 6 months after the start of the use of the Talkitt application.

##### 2.2.2.4. Follow-up

The primary outcome was the applicability and was assessed by means of a Satisfaction questionnaire at the end of the trial, i.e., 6 months after the onboarding phase. An intermediate measurement was carried out at the end of 1 month from the onboarding phase, to monitor the level of accuracy of recognition of the model and propose any technical adjustments. These results were not analyzed in the present study. The secondary outcome was the impact of the application use and was measured by the Adaptive Behavior questionnaire during the onboarding phase and at the end of the experimentation, after 6 months. Language abilities was also assessed at follow-up.

### 2.3. Measures

#### 2.3.1. Nonverbal intelligence

The Leiter-3 (Leiter et al., 2013) offers a nonverbal measure of intelligence and evaluates the ability to reason by analogy, matching and perceptual reasoning in general, irrespective of language, and formal schooling. This makes Leiter-3 particularly suitable for individuals with language difficulties. The nonverbal IQ obtained from the Leiter-3 is based on four subtests: Figure Ground, Form Completion, Classification and Analogies, and Sequential Order.

#### 2.3.2. Applicability: satisfaction questionnaire

The satisfaction questionnaire for the Talkitt device, filled by the user or the caregiver, consisted of 18 multiple-choice questions about the usability and frequency of use of the device. The following areas were investigated: quality of the instructions received for the use of the device, easiness and frequency of use, pleasantness of the interface, contents’ clearness, quantity of added words, the improvement of language production, and overall satisfaction were investigated.

#### 2.3.3. Language assessment

The Battery for Language Assessment in children aged 4 to 12 (Batteria di Valutazione Linguistica—BVL_4–12; Marini et al., 2015) systematically assesses phonological, lexical, semantic, pragmatic, and discursive skills in production, comprehension, and oral repetition tasks in children and adolescents, detecting communication and linguistic disturbances. This linguistic assessment scale comprises three sections: the assessment scale of oral production skills, the assessment scale of oral comprehension skills, and the oral repetition scale. To evaluate the articulation abilities of the participants and the intelligibility level of the speech, we used BVL_4–12, naming, and articulation subtests. Cronbach’s alpha are good for all age groups: mean values for naming range from 0.80 to 0.81, for articulation is 0.87.

#### 2.3.4. Adaptive behavior

The ABAS-II parent-report measure (Oakland, 2008) was used to assess the individual’s daily adaptive functioning. Parents or caregivers were asked to assess how often their child engages in a particular activity using a 4-item Likert scale (0—is not able, 1—never when needed, 2—sometimes when needed, and 3—always when needed). The measure consists of 10 skill areas: communication, community use, functional academics, home living, health and safety, leisure, self-care, self-direction, social, and work skills. ABAS-II provides norm-referenced standard scores for three domains: conceptual domain (CON), social domain (SOC), and practical domain (PRA) and a merged score—general adaptive composite (GAC)—(M 100, SD 15, and 90% and 95% confidence intervals and percentile ranks). Reliability coefficients for the general adaptive composite are in the high 0.90s for all age groups, ranging from 0.97 to 0.99. Reliability coefficients for the adaptive domains range from 0.91 to 0.98. Average reliability coefficients of the skill areas across age groups range from 0.85 to 0.97. Here, we provide some examples of the items included in the questionnaire: “Speaks clearly” (Conceptual domain), “carries scissors safely” (Practical domain”), and “says please when asking for something” (Social domain).

### 2.4. Statistical analyses

Descriptive statistics were used to describe the demographic characteristics of the participants. To evaluate the effect of Talkitt beta testing, repeated measure analysis of variance—ANOVA—has been performed on the ABAS II subscales composite scores and the BVL 4–12 tests raw scores, between T0 (before testing) and T1 (after 6 months of testing). The sphericity assumption, verified by Mauchly’s sphericity test, has been met. Bonferroni correction for multiple comparisons was applied so that the significant difference was set at the *p* < 0.0042 level. Partial eta squared (ηp2) was used to measure effect size. Outliers on the improvement, i.e., the difference between T1 and T0, were evaluated per each variable to identify if any participant benefited to a greater extent from the application. The Z-value was calculated, considering the mean and the standard deviation of the difference. The data with Z-values beyond 3 were considered as outliers. We identified only one outlier value: in particular, one participant showed a greater improvement in the ABAS Adaptive behavior Practical domain (Z-value = 3.9). To avoid any bias, this observation was dropped from the analyses.

### 2.5. Ethical considerations and data storage

The protocol was in full compliance with the Helsinki Declaration, and it was approved by the local Ethical Committee (163_OPBG_2018). Voiceitt manages a voice database of people with speech problems (“Impaired Speech Corpus”), an essential component to improve research and development of more advanced methods for recognition of speech impairment. Recordings of Talkitt users were continually copied to the database and the processes noted in the Library. No personally identifiable information has been transferred to this database. According to the new 2016/67 European Regulation, which entered into force on 25 May 2018, personal data, i.e., the e-mail address and voice recording, useful for using the Talkitt application or for monitoring technical problems, has been processed in the more absolute respect for the principles of correctness, lawfulness, relevance and non-surplus envisaged by art. Eleven of the aforementioned legislative decree, using IT tools, adopting suitable measures to guarantee the security and confidentiality of the data and will be kept for the time necessary and instrumental for the pursuit of the project’s purposes. Participants’ data are available only for the Data Processor and his collaborators. The participants’ personal data will not be disseminated.

## 3. Results

The accuracy of the application ranged between 60% to 95% according to the participant’s impairment. However, the results on the algorithm accuracy improvement will be analyzed as part of the broader project results and will be described elsewhere.

### 3.1. Application usability

**Figure 3:**
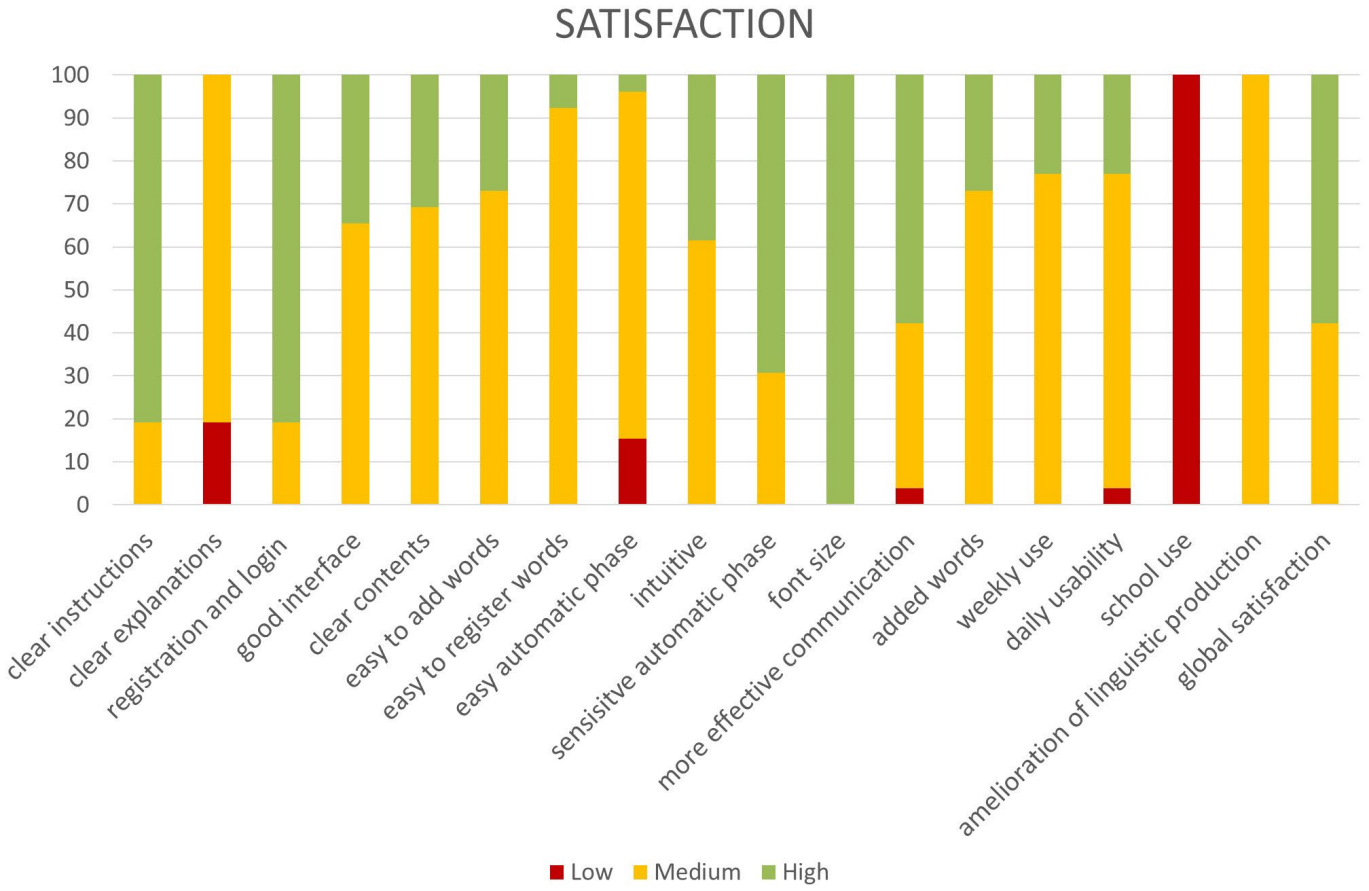
Parents report satisfaction with Talkitt use.

Application usability and functionality were assessed through a questionnaire collected from the parents. Data of the mean percentage of response are reported below (Figure 3).

As is shown, more than 70% of participants reported the highest level of satisfaction with: “Clear instructions,” “Easy registration and login,” and “Font size.” More than 70% of participants reported a medium satisfaction level for: “Easy word registration,” “Easy automatic phase,” “Used weekly,” “Daily usability,” and “Improves linguistic production.” Finally, more than 70% of participants reported the minimum level of satisfaction for: “Use at school.”

Of note, the management of the application was intuitive enough to require minimum or null support from caregivers.

### 3.2. Effect of Talkitt use on linguistic and adaptive functioning

Adaptive behavior was assessed by the ABAS II subscales composite scores after 6 months of Talkitt use. Although a general improvement was observed in all subscales, significant amelioration emerged in the Global composite score with a medium effect size. Similarly, a significant amelioration emerged in the verbal abilities, evaluated through the BVL 4–12 tests. Although both Naming and Articulation raw scores improved, only Naming improvement survived after Bonferroni correction and with a medium effect size (see Table 1 for details).

**Table 1:** Adaptive and linguistic measures at follow-up.

Test	Subscale	T0 mean	(SD)	T1 mean	(SD)	*F*(1,22)	*p*	*η* ^2^
ABAS adaptive behavior	Global^a^	55.6	14.2	62.4	17.0	10.78	0.003^*^	0.33
	Conceptual^a^	57.7	11.4	56.9	12.4	0.10	0.76	0.004
	Social^a^	71.4	15.8	76.4	17.9	4.18	0.05	0.16
	Practical^a,b^	56.2	17	60.5	19.7	8.75	0.008	0.29
BVL language production	Naming^c^	36.6	16.8	45.1	15.5	20.46	<0.001^*^	0.48
	Articulation^c^	30.1	29.3	40.0	35.2	4.71	0.04	0.18

^a^Standard score; ^b^*F*(1,21); ^c^row score.

* Significant after Bonferroni correction.

## 4. Discussion

This is the first study to report the satisfaction and the effects on linguistic abilities of an ML-based CAA intervention in children with DS and their caregivers who have participated in a 6-month beta test. The purpose of this work was to use Talkitt Application to train an Artificial Intelligence System in recognizing 20 unintelligible words/person in a sample of youths with DS. We also evaluated the caregivers’ satisfaction with using the Talkitt application and device. Finally, we investigated the possible benefits of adaptive behavior and language abilities deriving from the use of Talkitt application/device. The algorithm was trained correctly. Overall, caregivers of children who completed 6 months of the beta test were satisfied with the Talkitt application/device as a means to improve communication abilities. Caregivers perceived Talkitt as easy to use and beneficial to their children. The evaluation of language skills in our sample confirmed such perception, demonstrating an effective improvement of oral production in our sample. We also detected an amelioration of global adaptive abilities.

### 4.1. Talkitt has a good usability and exhibited high levels of satisfaction among caregivers

A majority of caregivers reported being very satisfied or satisfied with the following items: clearness of the provided instructions for the use of the device; the ease of registration and login; the pleasantness of the graphical interface; clearness of the provided contents; the ease in adding new words to train the algorithm; the easiness in registration new words; intuitiveness of the device/application; the sensitivity of the automatic recognition; the font size; the added words; the weekly use; the improvement of linguistic abilities; and overall satisfaction. Of note, the management of the application was intuitive enough to require minimum or null support from caregivers. Taken together, these results depict high levels of Talkitt usability and perceived usefulness. The delivery of an ML-based CAA intervention in children with DS is an innovative yet strategic approach, as it allows for overcoming barriers to interaction specific to this population. Automated speech analysis is a useful tool for analyzing and modifying speech in speech disorders, also in pediatric age (McKechnie et al., 2018). Children who need speech therapy could have significant barriers, given that this kind of intervention is often costly and time-requiring (McAllister et al., 2011) and caregivers could need alternative systems to gain access to services (Ruggero et al., 2012). Technology-based approaches can be elective tools to overcome these issues since they allow temporal and local independence, easy accessibility, and scalability (Ebert et al., 2018). Moreover, interventions based on the ML approach provide tailored support, helping to define the most appropriate course of action for a patient.

In particular, since Talkitt is an intelligent AAC solution, it may easily predict the language abilities of children with DS even if the input could be in part erroneous and incomplete. This aspect is of great advantage in comparison with the conventional classification methods (Thomas et al., 2017) because it can increase the probabilities of incoming words and phrases, and complete sentence transformations (Higginbotham et al., 2011) leading to a more proficient conversation.

Another advantage of intelligent AAC systems consists in the improvement and ease of use of devices and the associated user interfaces (Elsahar et al., 2019). The focus on the user activity to be carried out needs to be at the core of the implementation. Talkitt application shows high usability since its use does not require voluntary muscle controls, but the device is activated by the simple vocal recognition of the target words. This easiness of use could be of crucial relevance in cases of ID and possible difficulties in instruction understanding or executive functions (Costanzo et al., 2013).

Finally, affordability, in terms of costs associated with the hardware and software requirements of the utilized device, and portability, in terms of easiness of moving, have a great impact on the AAC device use (Elsahar et al., 2019). Since Talkitt is an available application, easy to download for different kinds of common-use devices such as smartphones or mini-tablets, it is suitable for usage in different settings.

However, the item concerning the use of Talkitt at school obtained the lowest score. In particular, caregivers referred to difficulties in verbal exchanges with classmates. Since the application was trained for a low number of words (about 20), which did not cover the entire vocabulary, it is possible that this could have limited the interactions in an unfamiliar environment.

Recently, the Voiceitt technology has evolved and uses a technology capable of updating itself more easily and of traslating entire sentences. Voiceitt’s next-generation technology recognizes “continuous” speech, i.e., vocabulary that has not been pre-calibrated. Surely, the use of an advanced level of the application could allow greater integration in environments outside the family and therapeutic one and could represent a valuable future development of the Talkitt application in DS.

### 4.2. Talkitt application/device improved adaptive abilities and language in children with DS

Talkitt application/device improved adaptive abilities in children with DS. Our results show a significant effect of the use of the Talkitt application/device on adaptive abilities, evaluated through ABAS II. Adaptive skills are defined as “the effectiveness with which the individual copes with the natural and social demands of his environment” (Heber, 1959). Thus, adaptive behavior supports autonomous functioning across several daily contexts and responsibilities (Tassé et al., 2016). In addition to impairment in cognitive and language abilities, children with DS exhibit important limitations in adaptive behavior. The limitation in adaptive behavior could be a direct consequence of reduced language abilities. Indeed, from an early age, children with developmental disabilities who have limited speech are strongly limited in participating in language and literacy instruction and social interaction; moreover, they are known to be at a greater risk for limited development of these skills for reasons both intrinsic to their disability and extrinsic to their learning environment (Ogletree, 2021). A recent review of the literature therefore highlights how AAC intervention can support not only vocabulary development and expressive language, but also social communication and adaptive behavior since preschool age (Allen et al., 2017; Ogletree, 2021). A possible explanation why general adaptive functioning may have improved after the application use could be related to a potential increase on participation, an essential dimension of human functioning according to the American Association of Intellectual Disabilities and Developmental Disabilities (Buntinx and Schalock, 2010). Participation includes social roles, involvement in leisure activities, choice, and control. A facilitation in communication exchanges by the application, may have indirectly affected the degree of participation and in turn the general adaptive functioning.

Although unexpected, we also found an improvement in linguistic abilities, in terms of speech and vocabulary improvement. The use of several different AAC systems (Binger et al., 2010; Kent-Walsh et al., 2010; Quinn et al., 2020) has been demonstrated to increase expressive vocabulary in children with DS. One possible reason could be that using AAC devices during a conversation would prompt the child to answer and elicit a response with the target word based on the AAC device, and that these strategies could support their vocabulary development (Ogletree, 2021). Given the positive outcomes that AAC application has had for vocabulary, language, and social communication development for children with DS, the use of AAC from a very early age seems promising. Moreover, given the high portability of the Talkitt device, i.e., the easiness of moving the device for usage in a different setting, its usability could be high for different contexts and different ages.

### 4.3. Limitations

However, this work is not without limitations. First, we evaluated only linguistic abilities at follow-up and no information is available on other cognitive measures. We cannot therefore exclude that the unexpected effect on naming improvement could be related to a general development or to other cognitive abilities changes. Further, it cannot be ruled out that the observed positive effects in naming abilities could have also positive effects on other cognitive domains; future studies are required to investigate these hypotheses. Finally, another limitation of the study is the lack of information on applicability (usability and satisfaction) and adaptive behavior impact from the participants themselves. This information is very important for understanding the individuals’ views and will need to be collected in future testing of new versions of the application.

Moreover, the use of the application did not exclude other concomitant treatments and was provided in addition to as usual activities. Another limitation of the study was not controlling for the type and amount of therapeutic and extracurricular activities of each participant. These aspects may have interacted with the effects of the app. It cannot be ruled out that the use of the application complemented with speech therapy could produce additional benefit on communication skills of children with DS. Future studies should take into account possible synergistic effects of the use of Talkitt plus concomitant treatment and activities.

Finally, there is a limitation of the present version of the application: the speech recognition is designed to recognize discrete words or phrases, for which the participant/user has provided adaptation data (or “seen data”) belonging to an individualized library. This limitation could lower spontaneity and fluency of the speech. To overcome this limitation, the algorithm should have the capability to recognize phrases for which the user has provided no data, or “unseen phrases.” Recent algorithmic developments of the app (Voiceitt next-generation technology) has evolved from discrete to continuous speech recognition, recognizing “continuous” speech, i.e., vocabulary that has not been pre-calibrated. This could lead to greater spontaneity and fluency without being limited to a closed vocabulary of pre-recorded phrases. This advanced version should be tested in future study to prove the accuracy and usability in population with DS.

### 4.4. Conclusion

These positive results and the high compliance emphasize the feasibility and efficacy of an ML-based AAC intervention for the improvement of communication abilities and adaptive abilities promotion for children with DS. Moreover, the advances in the integration of AAC systems with Artificial Intelligent applications could improve access to high-tech devices, the speed of output generation, and the customization and adaptability of the AAC interfaces to suit the needs and requirements of each individual user. Finally, the use of the present application could help expand the scope of AAC beyond physical communications, increasing the usability and the context of usage of future AAC solutions for children with DS.

## Data availability statement

The raw data supporting the conclusions of this article will be made available by the authors, without undue reservation.

## Ethics statement

The studies involving human participants were reviewed and approved by Bambino Gesù Children’s Hospital Ethical Committee. Written informed consent to participate in this study was provided by the participants’ legal guardian/next of kin.

## Author contributions

FC, SV, SS, and DW: conceptualization. FC, CC, SS, and DW: methodology. FC and CC: formal analysis. CC, DR, and FC: investigation. CC and DR: data curation. EF, FC, and CC: writing—original draft preparation. EF, FC, CC, SS, DW, and SV: writing—review and editing. SV: supervision and project administration. All authors contributed to the article and approved the submitted version.

## Funding

This work was supported by the EU’s Horizon 2020 programme with “SME Instrument Phase II: Talkitt” and by the Italian Ministry of Health with “Current Research” funds.

## Conflict of interest

DW and SS have a commercial and financial interest in the Talkitt application, since they are, respectively, CoFounder and CEO and Director of Business Development of Voiceitt.

The remaining authors declare that the research was conducted in the absence of any commercial or financial relationships that could be construed as a potential conflict of interest.

## Publisher’s note

All claims expressed in this article are solely those of the authors and do not necessarily represent those of their affiliated organizations, or those of the publisher, the editors and the reviewers. Any product that may be evaluated in this article, or claim that may be made by its manufacturer, is not guaranteed or endorsed by the publisher.
